# EARLY-LIFE CIRCUMSTANCES AND RACIAL DISPARITIES IN COGNITION AMONG US OLDER ADULTS

**DOI:** 10.1093/geroni/igad104.1011

**Published:** 2023-12-21

**Authors:** Zhuoer Lin, Justin Ye, Heather Allore, Thomas Gill, Xi Chen

**Affiliations:** Yale University, New Haven, Connecticut, United States; Yale University, New Haven, Connecticut, United States; Yale University, New Haven, Connecticut, United States; Yale School of Medicine, New Haven, Connecticut, United States; Yale University, New Haven, Connecticut, United States

## Abstract

There are marked racial disparities in cognitive outcomes between non-Hispanic Black (Black) and non-Hispanic White (White) older adults in the US. Existing studies concentrate on exploring mid-life to late-life risk factors, yet the early-life circumstances through which the racial gaps may arise remain under-explored. Using a wide spectrum of early-life factors assembled from the Health and Retirement Study, including demographics, SES, health, trauma, family relationships, and genetics, we investigated how racial gaps in cognitive score and cognitive impairment among older Americans were tied to racial differences in early-life circumstances. 9,015 (7,381 White, 1,634 Black) Americans aged 50 and older were included in the analysis. Using Blinder-Oaxaca-Decomposition, we showed that Black participants generally had less favorable early-life circumstances than White participants, especially in early-life residence, education level, quality and experience. We demonstrated that overall, differences in early-life circumstances explained up to 61.9% of the racial gaps in cognitive score, and 80.9% of the racial gaps in cognitive impairment between White and Black participants. Disentangling racial gaps by individual factors, we found that differences in the level and quality of education contributed the most. Years of education and school racial segregation respectively accounted for 22.7-24.8% and 26.0%-42.8% of the gaps in cognitive outcomes. Early-life educational experience and place of birth/residence additionally contributed to the disparities. However, childhood trauma, health, and genetic factors were not significant contributors. These findings suggest that exposure to less favorable early-life circumstances is associated with clinically meaningful and statistically significant racial gaps in cognition, which warrants targeted interventions.

